# Integrated human-animal sero-surveillance of Brucellosis in the pastoral Afar and Somali regions of Ethiopia

**DOI:** 10.1371/journal.pntd.0009593

**Published:** 2021-08-06

**Authors:** Rea Tschopp, Ashenafi Gebregiorgis, Yayehyirad Tassachew, Henok Andualem, Mahlet Osman, Mulugeta Waji Waqjira, Jan Hattendorf, Abdulkadir Mohammed, Muhammed Hamid, Wassie Molla, Simeneh Awoke Mitiku, Henry Walke, Maria Negron, Melissa Kadzik, Gezahegne Mamo

**Affiliations:** 1 Armauer Hansen Research Institute, Addis Ababa, Ethiopia; 2 Swiss Tropical and Public Health Institute, Basel, Switzerland; 3 University of Basel, Basel, Switzerland; 4 Hawasa University college of Medicine and Health Sciences, Hawassa, Ethiopia; 5 Debre Tabor University college of Medicine and Health Science, Department of Medical Laboratory,Debre Tabor, Ethiopia; 6 ALERT (All African Leprosy, Tuberculosis and Rehabilitation Training) Center Clinical laboratory, Addis Ababa, Ethiopia; 7 Addis Ababa University, College of Veterinary Medicine and Agriculture, Bishoftu, Ethiopia; 8 Samara University College of Veterinary Medicine, Samara, Ethiopia; 9 University of Gondor, College of Veterinary Medicine and Animal Sciences, Gondor, Ethiopia; 10 U.S. Centers for Disease Control and Prevention, Atlanta, Georgia, United States of America; Beijing Institute of Microbiology and Epidemiology, CHINA

## Abstract

**Background:**

Brucellosis is widespread in Ethiopia with variable reported prevalence depending on the geographical area, husbandry practices and animal species. However, there is limited information on the disease prevalence amongst pastoral communities, whose life is intricately linked with their livestock.

**Methodology:**

We conducted an integrated human-animal brucellosis sero-surveillance study in two adjacent pastoral regions, Afar and Somali region (SRS). This cross-sectional study included 13 woredas (districts) and 650 households. Blood samples were collected from people and livestock species (cattle, camel, goats and sheep). Sera were analyzed with C-ELISA for camels and shoats (sheep and goats), with I-ELISA for cattle and IgG ELISA for humans. Descriptive and inferential statistics analyses were performed.

**Results:**

A total of 5469 sera were tested by ELISA. Prevalence of livestock was 9.0% in Afar and 8.6% in SRS (ranging from 0.6 to 20.2% at woreda level). In humans, prevalence was 48.3% in Afar and 34.9% in SRS (ranging from 0.0 to 74.5% at woreda level). 68.4% of all households in Afar and 57.5% of households in SRS had at least one animal reactor. Overall, 4.1% of animals had a history of abortion. The proportion of animals with abortion history was higher in seropositive animals than in seronegative animals. Risk factor analysis showed that female animals were significantly at higher risk of being reactors (*p* = 0.013). Among the species, cattle had the least risk of being reactors (*p* = 0.014). In humans, there was a clear regional association of disease prevalence (*p* = 0.002). The older the people, the highest the odds of being seropositive.

**Conclusion:**

Brucellosis is widespread in humans and animals in pastoral communities of Afar and SRS with the existence of geographical hotspots. No clear association was seen between human and particular livestock species prevalence, hence there was no indication as whether *B*. *abortus* or *B*. *melitensis* are circulating in these areas, which warrants further molecular research prior to embarking on a national control programs. Such programs will need to be tailored to the pastoral context.

## Author summary

Brucellosis is a neglected disease of livestock that can be transmitted to people through consumption of raw animal products and direct contact with animal birth material. Although prevalent in Ethiopia, there is limited information on the disease prevalence amongst pastoral communities, whose life is intricately linked with their livestock. We therefore, conducted a disease surveillance in people and their livestock in Afar and Somali region (SRS), two pastoral regions of Ethiopia. The study included 13 districts and 650 households. A total of 5469 blood samples were collected from people and livestock species (cattle, camel, sheep and goats) and analyzed using commercial brucellosis ELISA assays. Livestock brucellosis prevalence was 9% in Afar and 8.6% in SRS (ranging from 0.6 to 20.2% at woreda level). In humans, prevalence was 48.3% in Afar and 34.9% in SRS (ranging from 0 to 74.5% at woreda level). 68.4% of all households in Afar and 57.5% of households in SRS had at least one positive animal. This study showed that the disease is widespread in human and their livestock in pastoral communities of Afar and SRS with the existence of geographical hotspots. Results will advise on tailored surveillance programs in pastoral communities.

## Introduction

Brucellosis is a significant, widespread contagious zoonotic bacterial disease, which has a substantial economic impact on the livestock sector. The disease is caused by a gram-negative, facultative intracellular bacterium from the genus *Brucella* which infects a wide range of animal species and humans [1–4]. Brucella species are not host specific but are known to have a host preference. For example, cattle are the main host for *B*. *abortus*, whereas *B*. *melitensis* is found primarily in small ruminants and *B*. *suis* in swine [5,6]. Among the many *Brucella* species isolated in humans and animals, *B*. *abortus*, *B*. *melitensis*, *B*. *suis* and *B*. *canis* are the ones classically described as the most important zoonosis [4,7].

In livestock, the disease is usually asymptomatic but may cause abortion storms in naive herds, infertility and decreased milk production [8–10], hence being responsible for considerable economic losses. In rare cases, joint inflammations are observed [11]. A peak shedding of bacteria occurs around the time of abortion or birth, which contributes to large environmental contamination [12–14].

Humans become infected through direct contact with fetal material, vaginal fluids and afterbirths from infected animals or through consumption of raw or undercooked meat or raw dairy products [13,15–17]. People with brucellosis often show nonspecific clinical signs and/or symptoms like malaise, fatigue, fever, muscle and joint pains [15,18–20] and possible spontaneous abortion in pregnant women [21,22]. The clinical presentation is often indistinguishable from other febrile diseases such as malaria and typhoid fever [15,23]. Chronic forms and recurrences can lead to long-term complications such as arthritis, endocarditis, spondylitis, recurrent fever and ME/CFS [24,25].

More than 500,000 human cases occur annually worldwide [26,27]. It is the second most important zoonotic disease in the world, next to rabies [28]. The disease has been controlled and/or eradicated in most developed countries, thanks to extensive control programs [7,28,29]. Brucellosis however, remains an important human-animal health and socio-economic problem in developing countries, where large rural populations rely mainly on their livestock for income and food [7,26,30] and where resources and coordinated control programs are lacking.

Ethiopia has the largest livestock population in Africa [31] as it is a major source of income and security for two-thirds of the population [32–34]. Brucellosis is endemic in Ethiopia, with a livestock prevalence ranging across geographical regions and livestock species from 3% to almost 50% [35]. Higher prevalence was found in some lowland and pastoral areas [36–40], but studies are very limited in scale. An estimated 40% of the livestock are kept in the pastoralist lowland areas [5,41]. The burden of human brucellosis is likely to be higher in these pastoral communities who have a cultural habit of consuming raw animal products, have daily physical contact with their livestock and often have poor access to health services.

Limited data is available on brucellosis prevalence at the livestock-human interface in pastoral areas [42]. This is the first large scale integrated animal-human serological surveillance of brucellosis in pastoral communities. The aims of this study were to quantify the seroprevalence of brucellosis in all livestock species and pastoralists alike as well as describe risk populations, using an integrated One-Health approach in two pastoral regions of Ethiopia, namely Afar and SRS.

## Material and methods

### Ethics statement

This study received ethical clearance in Switzerland from the “Ethikkommission Nordwest-und Zentralschweiz” (EKNZ) (R-2017-000666) and the institutional clearance at AHRI, Ethiopia (P041-17). Formal written consent was obtained from parents/guardians for child participants.

### Study site

**Fig 1:**
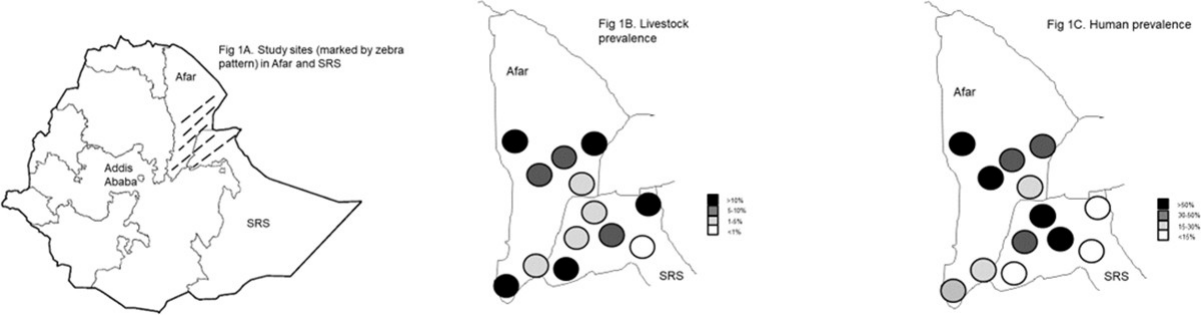
Map of Ethiopia showing the study sites (1A), the brucellosis prevalence in livestock (1B) and the prevalence in humans (1C). Map baselayer for all 3 maps is from: https://commons.wikimedia.org/wiki/File:Ethiopia_regions_blank.png.

The study was conducted between November 2017 and June 2018 in two neighboring pastoral regions of Eastern Ethiopia, Afar and SRS (Fig 1A). The regions are bordered to the East by Djibouti and Somaliland, to the South by Somalia and Kenya and to the North by Eritrea. The majority of the communities are pastoralists (90%), keeping livestock (goats, sheep, camels, and cattle) for their daily livelihood and social security. The climate is typically arid to semi-arid, and many places experience regular water and fodder shortages, forcing pastoralists to seasonal migrations with their animals. The study area is covered by sparse vegetation and extensive grazing land.

### Study design, study population and sample size

This cross-sectional study used a multistage cluster sampling proportional to size, according to Bennet et al 1991 [43]. Thirteen woredas (districts) were selected based on accessibility by road and safety, seven from Afar and six from the Somali region (SRS). In each woreda, kebeles (villages) were selected randomly with probability of selection proportional to the human population size. A total of 118 kebeles were selected and used as cluster units. Within each cluster, an official list of all households owning livestock was provided by the kebele chairmen and 650 households were randomly selected in which both, people and animals were tested. Within each selected household, all people over seven years of age were enrolled if they had provided informed consent to participate in the study. All livestock species were included (cattle, sheep, goat and camel) and were randomly selected in each household.

For the sample size determination, we assumed a prevalence of 6%, an intraclass correlation *rho* of 0.1 between clusters and a design effect of 4.9. A total sample size of 5000 samples were required for a precision estimation of 95% confidence limits around the estimate of 1.9%-points.

### Laboratory data

#### Specimen collection- animals & humans

Venous blood from livestock was drawn by trained health professionals into 10 ml Vacutainer labeled tubes, whereas 4 ml blood were used for humans. The blood samples were left at room temperature to allow clot formation. The serum was then carefully separated using sterile Pasteur pipettes into 1.5 ml labeled cryo-tubes and transported in a cool box with ice to the respective regional laboratory, where the serum as well as the remaining blood clots were stored in the freezer before being transported by air to the Armauer Hansen Research Institute, in Addis Ababa for further laboratory analysis and storage at -20°C. Animals are not vaccinated against brucellosis in Ethiopia.

#### Serological test- animals & humans

*Camel and small ruminant serum (sheep*, *goat) were analyzed using a commercial comparative* ELISA (SVANOVIR Brucella-Ab C-ELISA, Boehringer Ingelheim Svanova, Sweden). Serum from bovine were analysed by indirect ELISA (SVANOVIR I-ELISA-Ab, Boehringer Ingelheim Svanova, Sweden). Human samples were tested using an IgG ELISA (Demeditec Diagnostics, GmbH, Kiel, Germany). All laboratory procedures followed the manufacturer’s instructions.

A commercial immune-chromatographic lateral flow assay (LifeAssay Diagnostics (PTY) LTD, Cape Town, RSA) was used in people who refused to be tested by drawing venous blood or in addition to the conventional blood testing. The rapid test needed a blood drop from a finger prick and the results were read on the spot after a few minutes following the manufacturer instructions.

### Epidemiological data

A structured datasheet captured information related to the sampled person and animal. For people, information included demographic information (age, sex, address) as well as history of abortion. For animals, information included species, age, sex, and history of abortion.

### Data management and statistical analysis

All survey and laboratory data were entered, cleaned, and stored in Microsoft Access tables at AHRI. Statistical analysis was performed using STATA-15 Software (StataCorp, Texas, USA). Data analysis included both descriptive and inferential analysis. Generalized Estimating Equation (GEE) model for binomial outcomes was used to account for potential correlation within herds, to calculate apparent sero-prevalence in human and livestock populations and to examine the association between seropositivity and potential risk factors. Age was categorized depending on species. Age in human individuals was categorized as <20 years, 20 to less than 50 years (20-<50), and 50 years and older. Animals were divided into two age categories, those who had not reached sexual maturity and sexually mature adults (6 months for sheep and goat, 3 years for cattle and 4 years for camel).

A p-value <0.05 was considered to be significant. In animals, analyses were run by species, by livestock (all species regrouped) and by differentiating small ruminants (goats + sheep) and large animals (cattle + camel). Correlations across species were assessed using Spearman’s correlation coefficient for village level and household level prevalence.

True sero-prevalences (TP) were estimated using the Rogan–Gladen estimator:

TP = (AP + Sp—1)/(Se + Sp—1), where TP is the true prevalence, AP is the apparent prevalence, Se is the sensitivity and Sp is the specificity of the diagnostic tests [44]. Based on the manufacturer sensitivity and specificity evaluation documents, we considered Se = 100% and Sp = 98.78% for the human IgG Elisa; Se = 99.4% and Sp = 98.9% for C-ELISA in sheep and goats; Se = 97% and Sp = 100% for cattle I-ELISA. TP for camel was not done due to lack of evaluated data.

## Results

### Populations

In total, in both pastoral regions of Afar and SRS, 653 households were sampled (427 in Afar, 226 in SRS) among 118 villages in 13 Woredas.

### Human rapid test

In total, 410 rapid tests (RT) were performed, of which 352 (85.9%) were performed as sole test and 58 (14.1%) were done in addition to the ELISA test. All RTs were negative. Among the 58 participants with dual testing (RT + ELISA), 39 samples (67.2%) were ELISA negative. A further 17 samples (29.3%) were ELISA positive and two ELISA inconclusive.

### Sero-prevalence in humans and animals

Overall, 5469 sera were tested by ELISA, of which 3798 (69.4%) and 1671 (30.6%) were Afar and SRS samples, respectively.

In livestock, brucellosis prevalence was 9.% (N = 292/3202; 95%CI = 13.7–16%) in Afar and 8.6% (N = 130/1456; 95%CI = 9.9–13.8%) in SRS. Highest prevalence was found overall in goats (9.8%; 95%CI = 8.5–11.4), followed by sheep (8.3%; 95%CI = 6.4–10.6), camel (7.5%; 95%CI = 5.5–10) and cattle (7.1%; 5.2–9.7) (Table 1). Overall livestock prevalence ranged between 0.6% and 20.2% at Woreda level (Table 2).

**Table 1:** Sero-prevalence of brucellosis by region, detailed species and sex (using a GEE model).

Variable			Total tested	Total positive	Prevalence (%)	95%CI (%)
Region		Overall	5469	756	13.9	12.9–14.9
		Afar	3798	559	14.8	13.7–16.0
		SRS	1671	197	11.7	9.9–13.8
					
Species	Human	Overall	809	333	44.8	41–48.6
		Afar	594	266	48.3	43.9–52.7
		SRS	215	67	34.9	28.1–42.4
					
	Goat	Overall	2466	251	9.8	8.5–11.4
		Afar	1486	147	9.7	8.3–11.7
		SRS	980	104	9.5	7.3–12.3
					
	Sheep	Overall	856	71	8.3	6.4–10.6
		Afar	613	55	8.9	6.7–11.9
		SRS	243	16	6.6	4.0–10.6
					
	Cattle	Overall	604	43	7.1	5.2–9.7
		Afar	488	35	7.2	5.0–10.2
		SRS	116	8	6.9	3.5–13.2
					
	Camel	Overall	734	58	7.5	5.5–10.0
		Afar	617	56	9.0	6.6–12.2
		SRS	117	2	1.8	0.6–6.9
					
Sex	Human Female	Overall	410	161	42.0	37.0–47.1
		Afar	275	119	45.7	39.7–51.9
		SRS	135	42	33.8	25.5–43.2
	Human Male	Overall	399	172	45.7	40.6–50.9
		Afar	319	147	49.2	43.4–55.0
		SRS	80	25	32.2	22.9–43.2
					
	Livestock female	Overall	4209	397	9.2	8.2–10.4
		Afar	3010	278	9.1	8.0–10.4
		SRS	1199	119	9.4	7.4–11.8
	Livestock male	Overall	449	25	5.7	3.7–8.5
		Afar	192	14	7.3	4.3–12.0
		SRS	257	11	4.0	2.0–7.8

**Table 2:** Livestock and human prevalence by woreda (random effect on household/herd level).

	Human			Livestock			
Woreda	Total tested	Total positive	Prevalence (95%CI)	Total tested	Total positive	Prevalence (95%CI)	Number of livestock abortion (%)
Amibara	108	69	74.5 (64.2–82.7)	855	42	4.9 (3.4–6.9)	32 (3.7)
Awash	85	19	22.8 (14.4–34.3	390	48	12.1 (9.0–16.2)	24 (6.1)
Afambo	60	17	28.4 (18.3–41.2	230	8	3.5 (1.7–7.2)	11 (4.7)
Asayita	88	31	38.6 (27.6–50.8)	235	24	10.3 (6.7–15.3)	24 (10.2)
Chifra	90	49	55.8 (44.9–66.2)	566	89	15.7 (13.0–18.9)	65 (11.5)
Mile	78	53	72.2 (60.9–81.3)	404	35	8.5 (6.0–12.1)	19 (4.7)
Dubti	85	28	34.0 (24.5–45.1)	522	46	8.7 (6.3–12.0)	26 (5.0)
Afdem	40	14	37.4 (23.3–54.1)	257	7	2.7 (1.2–5.8)	4 (1.5)
Erer	38	22	63.6 (45.4–78.6)	282	9	3.2 (1.8–5.7)	2 (0.7)
Aysha	20	0	-	213	44	20.2 (13.3–29.5)	0
Shinile	35	24	66.8 (65.9–67.7)	246	16	6.5 (4.0–10.2)	1 (0.4)
Mieso	57	6	12.5 (5.2–27.1)	301	53	17.5 (12.7–23.6)	13 (4.3)
Hadegale	25	1	4.5 (0.6–25.8)	157	1	0.6 (0.09–4.3)	2 (1.3)

In humans, brucellosis prevalence was 48.3% (N = 266/594) in Afar and 34.9% (N = 67/215) in SRS. Sero-prevalence ranged between 0 and 74.5% at Woreda level (Table 2). In humans, the older the individuals the higher the prevalence (Table 3). The lowest and highest prevalence was observed in the age category below 20 years old (N = 30/103; 29.6.2%) and in people aged 50 and older (N = 74/145; 50.8%) respectively.

**Table 3:** Brucella tests results (rapid test and ELISA) in humans by age category.

Age category (in years)	Total people tested	Total RT (nb positive)	Total ELISA test (nb positive)	ELISA sero-prevalence (%)*
<20	195	88 (0)	103 (30)	29.6 (21.5–39.3)
20-<50	821	288 (0)	515 (229)	45.5 (41.1–50.0)
> = 50	180	34 (0)	145 (74)	50.8 (42.8–58.8)
Total	1196	410 (0)	763 (333)	

*Prevalence with random effect on household

There were 292 households (68.4%) in Afar and 130 in SRS (57.5%) respectively with at least one positive animal. At least one positive human reactor was found in 228 households in Afar (53.4%) and in 60 households in SRS (26.5%).

True seroprevalence calculations for humans, goat, sheep and cattle and its comparison with AP are shown in Table 4. Apparent prevalence and TP did not differ much in humans and bovines. In small ruminant, TP was a bit lower than the AP.

**Table 4:** Comparing apparent prevalence (AP) and true prevalence (TP) by species and region.

Species	Region	Sensitivity (%)	Specificity (%)	AP %	TP %
Human	Overall	100	98.78	44.8	44.11
	Afar			48.3	47.66
	SRS			34.9	34.09
Bovine	Overall	97	100	7.1	7.3
	Afar			7.2	7.4
	SRS			6.9	7.1
Goats	Overall	99.4	98.9	9.8	8.8
	Afar			9.7	8.7
	SRS			9.5	8.5
Sheep	Overall	99.4	98.9	8.3	7.3
	Afar			8.9	7.9
	SRS			6.6	5.6

**Fig 2:**
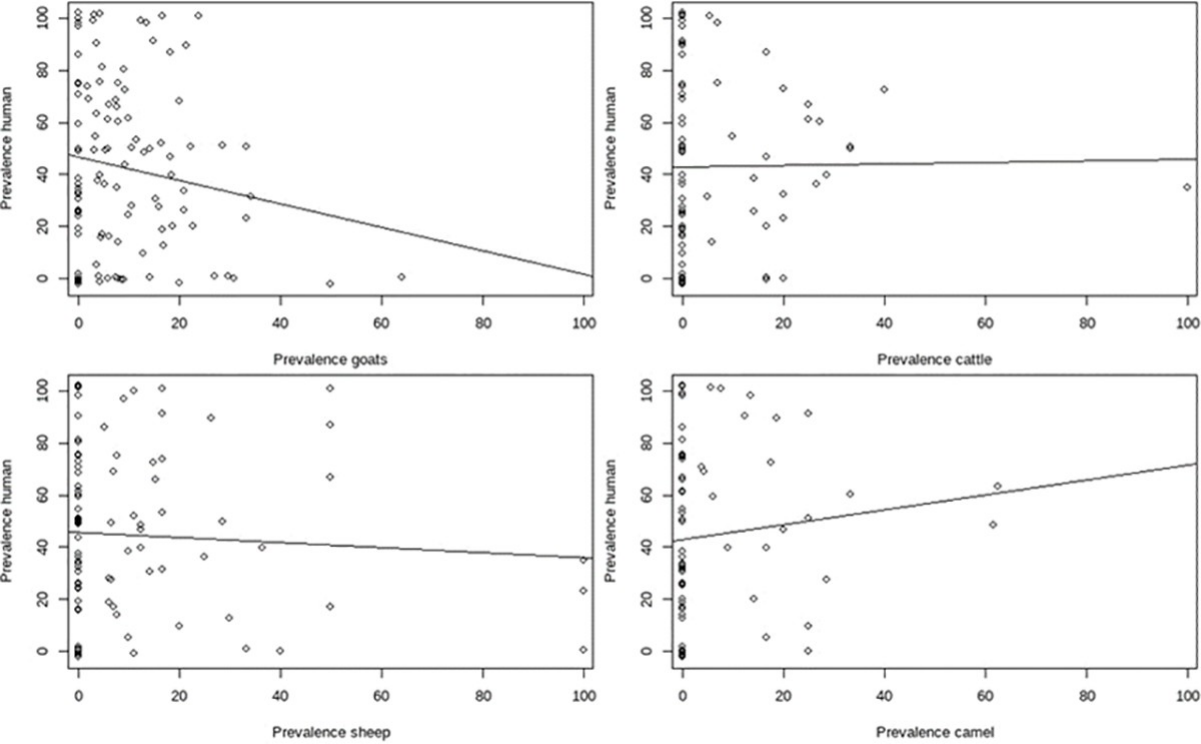
Correlation between animal and human sero-prevalence of Brucellosis at village level.

No correlation between species prevalence was observed at either household or village level (Fig 2).

### Abortions

Abortions were reported overall in 193 out of 4658 animals (4.1%) with a range between 0.0 and 11.5% at Woreda level (Table 2). The majority (85.0%) of livestock that aborted (N = 164/193) were ELISA negative. All cattle with abortion history were ELISA negative. A third of camels (N = 1/3; 33.3%), 16.0% (N = 21/132) of goats and 17.0% (N = 8/47) of sheep that had aborted were ELISA positive. Among the two women who had aborted in the last 12 months, one was ELISA positive for brucellosis.

The proportion of animals with a history of abortion was significantly higher (OR = 1.66; 95%CI = 1.08–2.54; *p* = 0.019) in seropositive animals compared to seronegative animals (Table 5). Table 5 shows the results between brucellosis status and abortion history in the different livestock species.

**Table 5:** Abortion history in relation to Brucellosis sero-status in livestock species.

Animal	Brucellosis status	Abortions	OR (95%CI)	*p*-value
Livestock	Neg	3.9% (164/4236)		
	Pos	6.9% (29/422)	1.66 (1.08–2.54)	0.019
Goat	Neg	4.9% (108/2215)		
	Pos	8.0% (20/251)	1.45 (0.85–2.44)	0.163
Sheep	Neg	4.7% (37/785)		
	Pos	11.3% (8/71)	2.4 (1.05–5.4)	0.038
Cattle	Neg	3.0% (17/561)		
	Pos	0/43	--	
Camel	Neg	0.3% (2/676)		
	Pos	1.7% (1/58)	6.4 (0.6–69.4)	0.125

### Univariate and multivariate analysis

We assessed in livestock five potential risk factors for brucellosis sero-positivity. Overall, in livestock, no significant association was observed in the univariate analysis with the region and with age (Table 6). Males were at significantly lower risk of being seropositive than females (OR = 0.59; *p* = 0.013). Among the species, cattle had less risk of being sero-positive than goats (OR = 0.63; *p* = 0.011). All variables were included in the multivariate model. Results from the multivariate analysis were similar. Univariate analysis done for each separate species showed that statistical significance was found in goats for males being protective (OR = 0.45; 95%CI: 0.25–0.81; *p* = 0.008) and in sheep with abortion history (OR = 2.25; 95%CI:0.97–5.18; *p* = 0.057).

**Table 6:** Univariate analysis in livestock with random effect on household/herd level (GEE model).

Variable		Number positive/total (%)	OR	95%CI for OR	SE*	*p*-value
Region	Afar	292/3202 (9.1)				
	SRS	130/1456 (8.9)	0.96	0.73–1.25	0.13	0.785
Woreda	Amibara	42/855 (4.9)				
	Awash	48/390 (12.1)	2.67	1.64–4.35	0.66	0.000
	Afambo	8/230 (3.5)	0.70	0.30–1.61	0.29	0.407
	Asayta	24/235 (10.3)	2.22	1.24–3.94	0.65	0.007
	Chifra	89/566 (15.7)	3.61	2.32–5.61	0.81	0.000
	Mile	35/404 (8.5)	1.81	1.08–3.04	0.47	0.024
	Dubti	46/522 (8.7)	1.84	1.12–3.02	0.46	0.015
	Afdem	7/257 (2.7)	0.53	0.21–1.31	0.24	0.174
	Erer	9/282 (3.2)	0.63	0.28–1.41	0.26	0.267
	Aysha	44/213 (20.2)	4.99	3.00–8.29	1.29	0.000
	Shinile	16/246 (6.5)	1.3	0.7–2.60	0.45	0.354
	Mieso	53/301 (17.5)	4.11	2.53–6.67	1.01	0.000
	Hadegale	1/57 (0.6)	0.12	0.01–1.07	0.13	0.059
Age	Young (not breeding)	13/201 (6.5)				
	Adult (breeding age)	409/4457 (9.2)	1.38	0.78–2.42	0.39	0.258
Species	Goat	251/2465 (10.2)				
	Sheep	71/856 (8.3)	0.83	0.63–1.10	0.11	0.212
	Cattle	42/603 (6.9)	0.63	0.44–0.89	0.11	0.011
	Camel	58/734 (7.9)	0.76	0.56–1.03	0.11	0.085
Sex	Female	397/4209 (9.4)				
	Male	25/449 (5.6)	0.59	0.38–0.89	0.12	0.012

*SE = Standard Error

In humans, three risk factors were assessed for individual human seropositivity (Table 7). A significant association was observed with region with SRS being less of a risk for sero-positivity than Afar (OR = 0.57, *p* = 0.002). There was no significant difference observed between male and female and sero-positivity. The older the people, the highest the odds of being sero-positive (OR = 1.93 for age category 20-<50 years and OR = 2.44 for people aged 50 and older). Similar results were found in the multivariate analysis.

**Table 7:** Univariate analysis in people with random effect on household (GEE model).

Variable		Number positive/total (%)	OR	95%CI for OR	SE	*p*-value
region	Afar	266/561 (47.4)				
	SRS	67/202 (33.2)	0.57	0.40–0.81	0.10	0.002
Age	<20 years	30/103 (29.1)				
	20-<50 years	229/515 (44.5)	1.93	1.26–2.95	0.41	0.002
	= >50 years	74/145 (51.0)	2.44	1.47–4.04	0.62	0.000
Sex	Female	161/386 (41.7)				
	Male	172/377 (45.6)	1.11	0.84–1.45	0.15	0.447

## Discussion

This large-scale study integrated animal and human surveillance of brucellosis within the same households in two pastoral regions of Ethiopia. Such a One-Health approach is important in assessing zoonosis such as brucellosis, particularly among pastoral communities, whose way of life is intricately linked with their animals. This study complements a national brucellosis surveillance that focuses on sedentary farmers mainly on the Highlands and the Southern regions of Ethiopia and will provide the Ethiopian Government with data needed for future control programs of brucellosis.

Pastoral areas are often neglected when it comes to disease surveillance and health services, due to the remoteness of the areas, challenging logistics, harsh environment, lack of infrastructure and sometimes security issues. The Afar and the SRS however, are two important pastoral regions in Ethiopia in terms of size, livestock herds, livestock economics and human and animal cross-border movements within the Horn of Africa. These regions thus, need special attention and inclusion in national surveillance programs.

Brucellosis was found to be widespread in the study areas, with over half of the households/herds having at least one positive case (68.4% in Afar and 57.5% in SRS). Livestock prevalence was 9% in Afar and 8.6% in SRS. The results are a bit higher than previously reported from these regions [34,45]. Comparison of results with previous studies performed should however, be cautiously done since various diagnostic methods are used in the different studies (e.g. RBP, ELISA, CFT). Also, few studies reported combined sero-prevalence results with consideration of all four species common to pastoralists (cattle, goat, sheep and camel), making comparison between studies more difficult. Nevertheless, our results were comparable to previous reports of 10.6% and 9.6% sero-prevalence of brucellosis in cattle and camels of Borena zone and SRS, respectively [46,47]. Although exposure to the disease in animals was observed in all woredas ranging from 0.6% to 20.2%, the sero-prevalence was not evenly distributed throughout the region and rather showed hotspots for disease exposure. The highest animal prevalence was found around Central Afar (Chifra), the areas bordering the Oromia region (Awash; Mieso) and Djibouti (Asayita, Aysha). The lowest prevalence was found along the Afar-SRS border and further south into SRS (Fig 1B). In comparison, antibody detection in humans was significantly higher in Afar (48.3%; *p* = 0.002) than in SRS (34.9%). Overall, high exposure levels (up to 74.5%) were seen in pastoralists (Fig 1C). Human exposure to brucellosis was higher than previously described in pastoral areas of Borena in Southern Ethiopia and in Amibara district [23,48]. In humans, there was no difference in risk of being sero-positive between male and females but there was an increasing risk with increasing age. Obviously, the chance of being exposed with the pathogen is increasing the longer one lives. The ELISA diagnostic for humans was targeting IgG, which can persist for many years as opposed to IgM and can be indicative for past infections [49]. Interestingly, no clear association could be seen between the current livestock prevalence and human prevalence. Human sero-positivity was not highest in areas with high animal sero-positivity (Fig 1B and 1C). Furthermore, 165 households with sero-positive people had no sero-positive livestock supporting the hypothesis that people might have been infected once during their life by their livestock, but by livestock no longer in their herd, or from products originating from other livestock (e.g. neighbor, family, market) showing that brucellosis has been circulating for a while in those areas. Livestock on the other hand, particularly goats and sheep, unlike people have a short life span and high herd turn-over.

Surveillance of zoonotic diseases in pastoral communities can be challenging for various reasons: accessibility, lack of diagnostic facilities, remoteness, difficulty maintaining the cold chain for samples and sometimes unwillingness to have venous blood drawn, to name some of the constraints. A rapid test as the one used in our study would be attractive for such settings. It does not need a cold chain, can be done with a simple finger prick and results read on the spot. The test was usually well accepted by the community. In rare cases, people refused to be tested for fear that it was a HIV test. Poor performance of this test was observed in Kenya’s health facilities in a low-epidemic setting [50]. On the opposite, good results were observed in previous studies in Southern Ethiopia [48]. However, in our study, none of the people tested by RDT were sero-positive although 29.8% of these were ELISA positive. Unlike the IgG ELISA, the RDT detected IgM. Hence, there is the possibility that it could not capture older infections in people. This rapid test, however, could prove useful for health clinics in high brucellosis prevalence areas, where patients are presented with brucella-like illness.

Brucella is known for easily crossing species barriers [51]. In our study, brucella seropositivity was found in all livestock species. Interestingly, although most (81.0%) pastoralists keep several livestock species, the disease did tend to affect only one of them. Indeed, 200 households had only one species showing sero-positivity, 46 households had two specie whereas only three households had all their livestock species affected. In total 150 households kept all fours livestock species. This could be explained by the fact that pastoralists often herd the various livestock species separately during the day and also keep the species separate during the night. This would reduce the risk of cross-species infection in households.

So far, few studies have isolated and identified *Brucella* species in Ethiopia. *Brucella abortus* was isolated in dairy cattle [52], whereas *B*. *melitensis* was isolated in goats in Amibara woreda (Afar) [53]. Although all livestock species were affected, it is at this stage difficult to conclude whether there is cross-species transmission of either *B*. *abortus* or *B*. *melitensis* or if both pathogens are equally circulating in the areas. More research is warranted in these pastoral areas to determine the prevalent *Brucella* strains, which will also be essential knowledge before embarking on any vaccination program.

Apparent overall sero-prevalence was 9.8% in goats, 8.3% in sheep, 7.1% in cattle and 7.5% in camels. Cattle were significantly less at risk of sero-positivity than goats (OR = 0.63; *p* = 0.011). In Southern Ethiopia, Gumi et al (2013) found similar sero-prevalence in goats (9.6%) in Oromo and Somali pastoralist communities but much lower sero-prevalence in cattle (1.4%) and camels (0.9%). Another older study from Afar and SRS showed a prevalence of 13.2% in goats and 5.6% in sheep and a difference between Afar (13.2%) and SRS (1.9%) in shoat brucellosis prevalence [54]. In the meta-analysis done by Tadesse (2016), sero-prevalence of brucellosis was 8.4% in shoats in Afar and 2.6% in SRS; camel prevalence was 4.8% in Afar and 2% in SRS. Similarly, Ibrahim et al (2020) showed low sero-prevalence of Brucellosis in SRS [55]. Based on our results and previous published results from these two regions [34,47,55], we hypothesize that SRS has a lower brucellosis sero-prevalence in livestock but that our study area in SRS (Sitti zone), is bordering Afar and hence this particular zone might have a higher sero-prevalence than the rest of SRS.

Overall in livestock, males had lower sero-positivity than females (OR = 0.59; *p* = 0.012). This is a tendency often observed [34]. It must be noted that females in our study represented 90.4% of all animals. Generally, pastoral herds comprise more females than males. Female animals are kept longer as milk providers and have thus increased chance of exposure to the pathogen, whereas males are used mainly for income or meat (selling or slaughtering). But this fact also raises the zoonotic risk as pastoralists will consume raw milk from these female animals. In our study, age was not significantly associated as a risk factor. However, caution must be taken with the result, since we did not have pre-puberty tested animals in shoats.

Brucellosis causes abortions particularly in naïve livestock herds and/or first pregnancy, impacting household economies and putting pastoralists livelihood at risk. None of the cattle that had aborted were seropositive for brucellosis suggesting other causes for abortions in this species. On the other hand, a third of the camels that had aborted were sero-positive for brucellosis. The proportion of animals with a history of abortion (except for cattle) was much higher in sero-positive animals than in sero-negative animals.

In conclusion, this wide scale integrated surveillance showed that brucellosis is endemic in pastoral communities in East Ethiopia with hot-spot areas that would need attention from the public and animal health authorities, particularly in light of the high sero-prevalence found in people. National surveillance and control programs have to include these remote pastoral communities but will likely need to be tailored to the particular context of pastoralism.
